# A Modified Direct Lateral Approach for Lateral Border and Scapular Spine Fixation in Floating Shoulder

**DOI:** 10.7759/cureus.88478

**Published:** 2025-07-21

**Authors:** Mohd Afiq Muhamed Fuad, Kamarul Ariffin, Ashraf Hakim Ab Halim

**Affiliations:** 1 Orthopaedic/Advance Musculoskeletal Trauma, Faculty of Medicine and Health Sciences, Universiti Putra Malaysia, Serdang, MYS; 2 Orthopaedics, Hospital Sultan Idris Shah, Selangor, MYS

**Keywords:** floating shoulder surgery, minimal invasive approach, modified lateral approach, scapular fractures, upper extremity trauma

## Abstract

Floating shoulder injuries are rare but require careful management to ensure functional recovery. Traditional approaches for scapular fixation often necessitate multiple incisions. We present a modification of the direct lateral approach that allows for scapular lateral border and scapular spine fixation using a single incision with utilization of subdeltoid space.

A 48-year-old male patient presented with a displaced extra-articular scapular fracture associated with a midshaft clavicle fracture following a motor vehicle accident. The patient underwent open reduction and internal fixation (ORIF) using a standard anterior approach for the clavicle and a modified direct lateral approach for the scapula. This technique eliminated the need for a secondary incision while ensuring stable fixation and preserving deltoid origin without the need for detachment. Postoperatively, the patient demonstrated excellent range of motion without complications up till his one-year follow-up. This case highlights a less invasive yet effective technique for scapular fixation in floating shoulder injuries.

## Introduction

Floating shoulder injuries, defined as concomitant fractures of the clavicle and scapular neck or body, are rare and account no more than 1% of all fracture, but represent a serious disruption of the superior shoulder suspensory complex (SSSC) that suspends the upper limb from the axial skeleton and is composed of the glenoid process, coracoid process, coracoclavicular ligament, clavicle, acromioclavicular joint, and acromial process [1]. These injuries are often caused by high-energy trauma, such as motor vehicle accidents or falls from height, and are associated with significant functional impairment such shoulder dysfunction and visibly drooping shoulder if not properly treated. Due to their complexity and rarity, the optimal surgical strategy for floating shoulder remains debated [1].

Traditional approaches for scapular fixation, such as the Judet and reverse Judet approaches, typically require multiple incisions to adequately expose both the scapular lateral border and spine. While effective, these methods often involve extensive soft tissue dissection, which may increase operative time, blood loss, and postoperative morbidity [2,3]. To minimize these issues, several modified techniques have been introduced with a focus on reducing surgical invasiveness while maintaining adequate fracture fixation [4].

In this report, we describe a modification of the direct lateral approach that permits fixation of both the scapular lateral border and scapular spine through a single incision. This technique utilizes the subdeltoid space for improved exposure while preserving the deltoid origin, potentially reducing surgical morbidity and improving outcomes in selected patients with floating shoulder injuries.

## Case presentation

**Figure 1:**
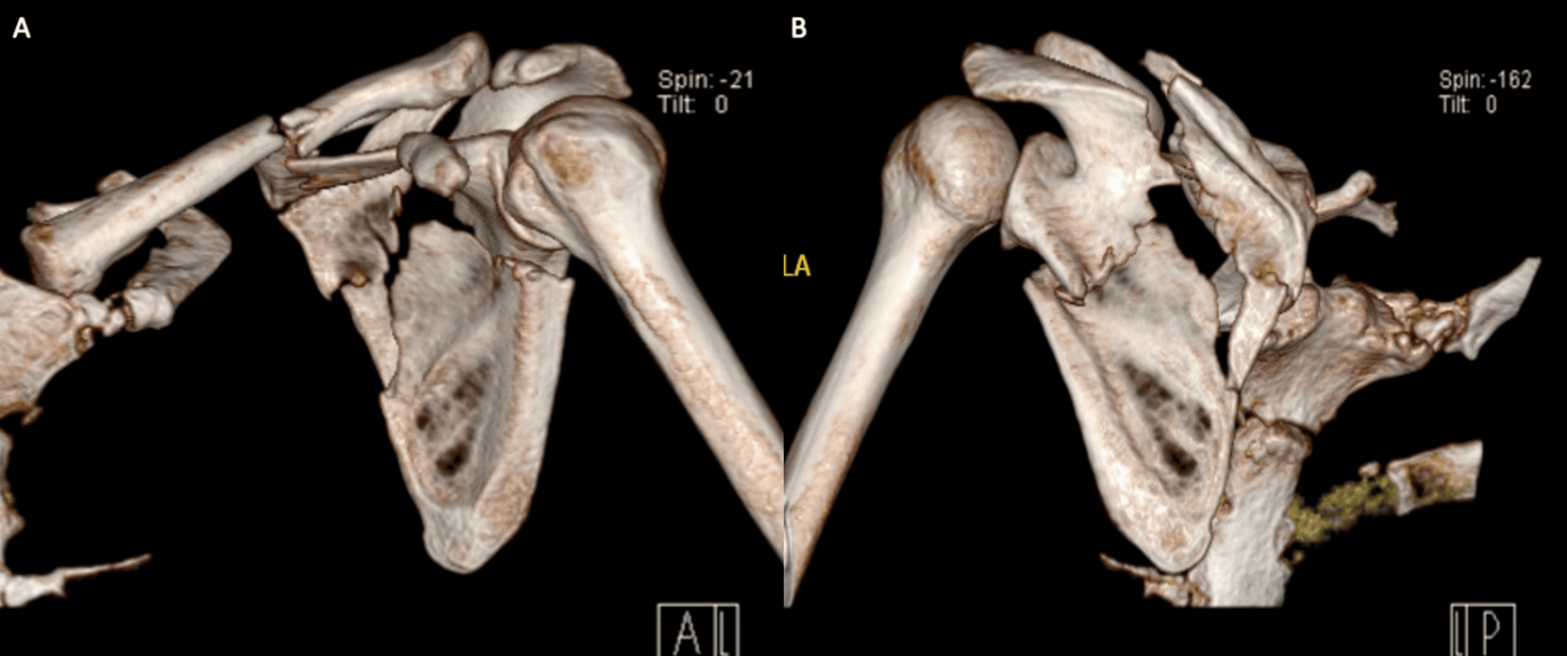
Preoperative 3D CT A: 3D CT of left scapula (anterior aspect) B: 3D CT of left scapula (posterior aspect)

A 48-year-old male patient presented with left shoulder pain following a motor vehicle accident. Clinical examination revealed localized tenderness over the left scapular region without neurovascular compromise. Radiographs and CT scans demonstrated a displaced extra-articular scapular fracture originating from the infra-glenoid and extending above the scapular spine, with an associated midshaft clavicle fracture (Figure 1) type B3 based on classification of displaced fracture of the scapula with a stable shoulder girdle [5]. The patient opted for surgical intervention.

**Figure 2:**
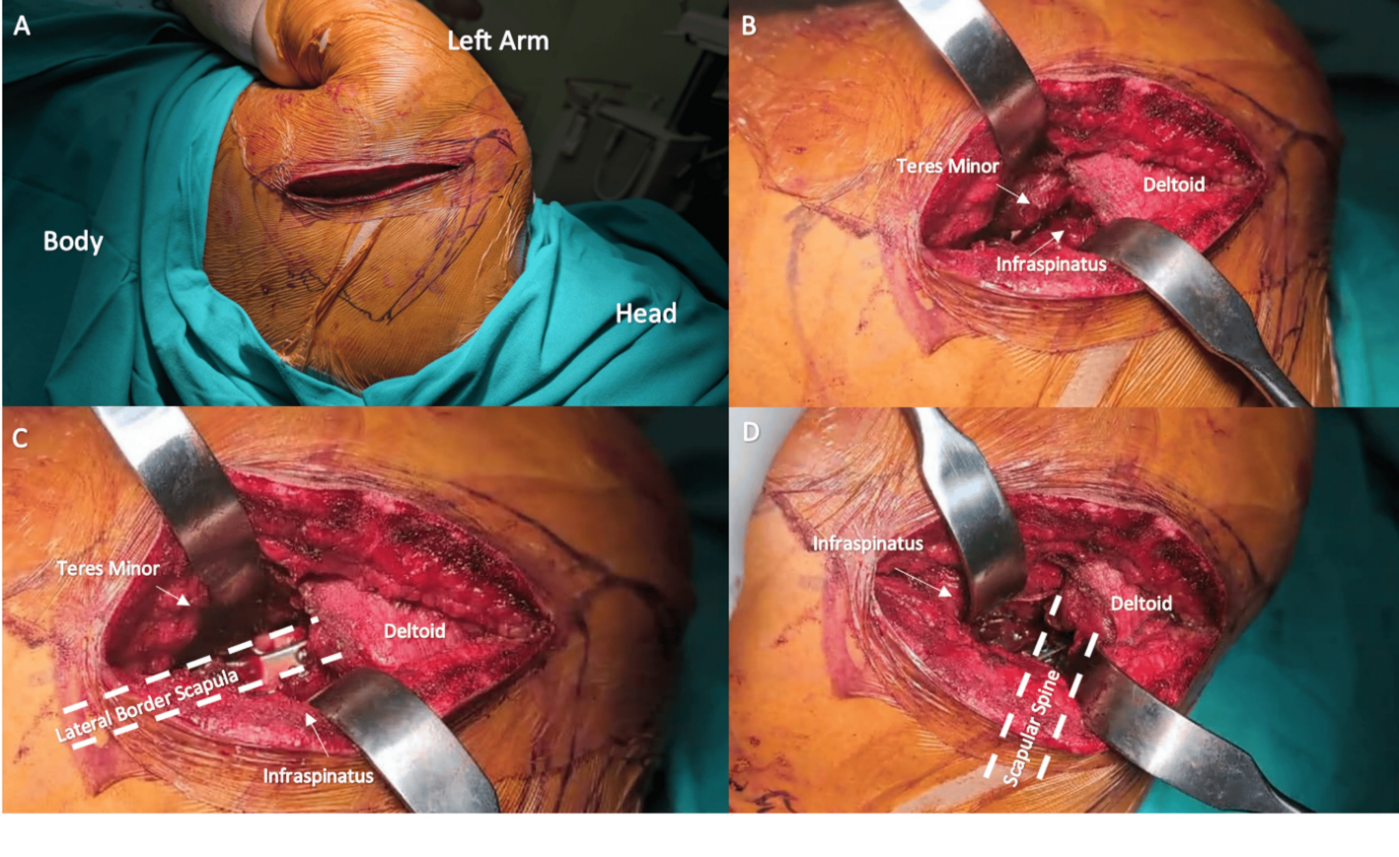
Intraoperative findings A: The incision made along the lateral border of the scapula B: Identification of the lower border of deltoid muscle and inter-nervous plane of teres minor and infraspinatus C: Retraction of the infraspinatus and teres minor to give the window for lateral border D: Elevation of the deltoid without releasing it from it attachment and retraction of the infraspinatus muscle inferior-laterally to create the second window that will give access to the scapular spine White dotted line: Surgical window created for fixation

The patient was positioned on his side, with the affected limb supported on a specialized frame. The clavicle fracture was initially stabilized through a conventional anterior approach, using an anatomical locking plate. For the scapula, a single incision was made along the lateral border, following an imaginary line drawn from the glenoid to the inferior angle of the scapula. The deltoid fascia was identified, and the dissection continued through the inferior border of the deltoid muscle. The deltoid was then retracted laterally and superiorly to expose the infraspinatus and teres minor muscles. The fascia separating these muscles was incised, and the lateral border of the scapula and glenoid neck were further exposed by retracting the infraspinatus superior-medially and the teres minor inferior-laterally. The ascending branch of the circumflex scapular vessels at the inferior border of the glenoid neck was identified and ligated. The fracture along the lateral border of the inferior glenoid neck was reduced using a small point reduction clamp, and definitive fixation was achieved using 2.7 mm plates. Then, deltoid muscle was further elevated superiorly and subdeltoid space was further utilized to allow exposure to the scapular spine without needing to detach the deltoid origin (Figure 2). 

**Figure 3:**
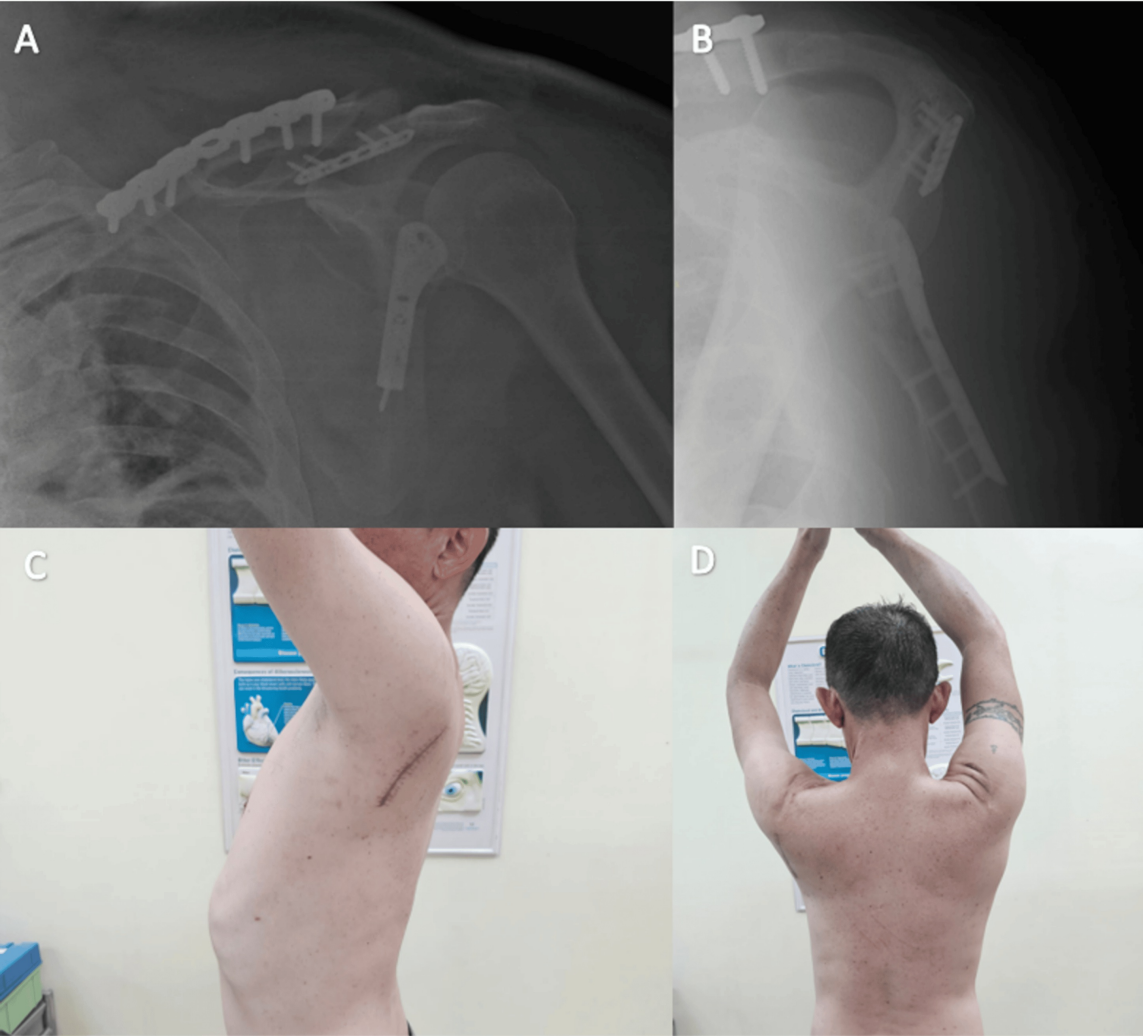
Postoperative radiograph and shoulder range of motion A: Radiograph (anterior-posterior view) B: Radiograph (scapula-Y view) C: Full flexion postoperative six months D: Full abduction postoperative six months

The 2.7 mm straight locking plate were used to help with reduction and before completion of the fixation over the scapular spine. The position of the fixation was confirmed using intraoperative imaging. Total operative time was 120 minutes, and the estimated blood loss was 300 ml. The patient had an uneventful postoperative course. The surgical wound healed well by the second postoperative week. A structured rehabilitation protocol was initiated, including immediate pendulum exercises postoperatively, passive range of motion exercises assisted by the contralateral hand, and progressive resistance exercises commencing at one month postoperatively. Three months postoperatively, the patient achieved a full range of motion, including shoulder abduction and flexion from 0° to 180°, with a Disabilities of the Arm, Shoulder and Hand (DASH) score of 3.3 at six months (Figure 3) [6]. No complications were noted during the one-year follow-up period.

## Discussion

A "floating shoulder" injury describes a combined fracture of the clavicle and scapula on the same side. More precisely, it involves a double disruption of the SSSC, a ring of bone and soft tissue that connects the arm to the body. This complex includes the glenoid, coracoid process, distal clavicle, acromioclavicular joint, acromion, and the coracoclavicular ligaments [1]. Floating shoulder injuries are uncommon but can result in significant instability if not properly managed [2].

Surgical stabilization is often required for floating shoulder injuries to restore biomechanics and prevent long-term functional impairment. The reverse Judet approach is a well-described technique for accessing the lateral border of the scapula [3]. However, this approach typically necessitates a separate incision to address the scapular spine, increasing surgical invasiveness and soft tissue disruption.

Several surgical approaches have been described for scapular fractures, with the choice depending on the fracture location and type. The Judet approach allows posterior exposure of the scapula, including the glenoid and body, but requires extensive muscle dissection, particularly of the infraspinatus, which can lead to increased morbidity [4]. To reduce soft tissue disruption, the modified Judet approach was developed. This approach retains the same skin incision but minimizes muscle dissection, preserving infraspinatus function. It provides complete joint exposure for intra-articular fractures, better access to the scapular body and neck for fixation, and lower morbidity compared to the traditional Judet approach [4].

The vertical approach, as described by Hardegger et al., involves a vertical incision from the acromion to the inferior scapular angle [7]. However, posterior deltoid release is not possible, making visualization of high glenoid neck fractures difficult. In contrast, the direct lateral approach, as reported by Mannambeth et al., offers a minimally invasive surgical technique for stabilizing displaced extra-articular scapular neck and body fractures [8]. This approach focuses on plating the lateral column of the scapula through a targeted incision, utilizing the interval between the infraspinatus and teres minor.

Our approach utilizes the direct lateral approach concept to provide stable and rigid fixation over the lateral body of the scapula and the infraglenoid area. The modification in this approach involves further utilizing the subdeltoid space to create a surgical corridor for scapular spine plate placement, providing additional stabilization to the fixation. However, the angle of the screw trajectory that almost parallel to the patient body for the scapular spine remains the same as in other approaches. In a case with severe displacement of the scapular spine despite scapular spine fixation, a surgeon might encounter difficulty in using a reduction clamp in this modified approach. In such a case, using a plate reduction technique might ease the process. The goal of this approach is to achieve adequate exposure and stabilization of the scapular fracture while minimizing soft tissue disruption and preserving shoulder function. A more comprehensive study would help establish the effectiveness of this approach.

## Conclusions

A modified direct lateral approach provides an effective, minimally invasive solution for scapular fixation in floating shoulder injuries. By avoiding additional incisions, this technique reduces surgical morbidity while ensuring stable fixation. This case highlights the potential for refining surgical approaches to optimize patient outcomes. The preservation of deltoid origin and utilization of subdeltoid space can be used to improved access to the scapular spine without additional dissection represents a notable advancement. This technique may serve as a valuable alternative in future cases where traditional approaches are less favorable.
